# Genetics in the X-Men film franchise: mutants as allegories of difference

**DOI:** 10.3389/fgene.2023.1331905

**Published:** 2024-01-03

**Authors:** Sonora R. Grimsted, Katerina G. Krizner, Cynthia D. Porter, Jay Clayton

**Affiliations:** ^1^ Divinity School, Vanderbilt University, Nashville, TN, United States; ^2^ Department of English, Vanderbilt University, Nashville, TN, United States; ^3^ Department of Germanic Languages and Literatures, The Ohio State University, Columbus, OH, United States

**Keywords:** X-Men, comic book movies, Marvel Cinematic Universe, othering, genetic difference, mutants, chosen families

## Abstract

This article analyzes the complete corpus of live-action X-Men movies for their depictions of genetics and otherness. The researchers watched and qualitatively coded all thirteen movies produced by 20th Century Fox that take place in the same shared cinematic universe, beginning with *X-Men* (2000) and ending with *The New Mutants* (2020). The X-Men movies are unusual summer blockbusters since they explore genetic topics through their central characters, mutants, who are genetically different from their non-mutant peers. Mutants in the films evoke a plurality of analogies, such as mutant-as-Black and mutant-as-queer. These intersecting metaphors build upon a core of genetic difference to create a versatile but limited picture of prejudice, solidarity, and otherness.

## Introduction

I think the American people deserve the right to decide whether they want their children to be in school with [those people], to be taught by [them]. Ladies and gentlemen, the truth is that mutants are very real, and they are among us.^1^

We have people that live among us today on planet Earth that are happy to display themselves as if they were mutants from another planet.^2^

The first quote is from a fictional U.S. senator in the movie *X-Men* (2000), speaking about a potential mutant registration bill that would require all mutants to register themselves with the federal government. The second quote is from a real-life Republican state congressman in Florida in 2023 speaking about a potential “bathroom bill” that would limit the right of trans people to use the bathroom of their choice (Atterbury, 2023). These quotes, one fictional, the other real, are separated by more than two decades, but they could easily be part of the same speech.

Both lawmakers demonstrate an aversion to “those people,” people unlike themselves. They worry that the Other is lurking “among us,” the presumed normal majority. For Representative Barnaby the worrisome Other is trans people, but similar comments have been made about Jews, African Americans, and countless other minoritized groups at different moments in history. Mutants of the X-Men movies do not map directly onto any of these real-world populations, but they do present a compelling picture of an othered group in popular cinema.

The X-Men films, based on the X-Men comic books, are big-budget, widely viewed, mainstream movies that are full of glib talk about genetics and powerful depictions of bias against othered populations. There are thirteen films that take place in the X-Men universe, all produced by 20th Century Fox between 2000 and 2020: *X-Men*, X2 (2003): *X-Men United*, X-Men: Last Stand (2006), *X-Men Origins: Wolverine*, *X-Men: First Class*, *The Wolverine, X-Men: Days of Future Past*, Deadpool (2016), *X-Men: Apocalypse*, *Logan*, *Deadpool 2*, *X-Men: Dark Phoenix,* and *The New Mutants*. The X-Men movies are joined by many more films in the well-known Marvel Cinematic Universe (MCU) produced by Marvel Studios (now a subsidiary of Disney), which has only increased the visibility of Marvel franchises like X-Men at other studios. Fox’s X-Men franchise is considered a commercial success with a collective international box office total of 3.6 billion dollars, despite their uneven reputation among critics. Some have received favorable critical responses, such as *X-Men: Days of Future Past* (90% Rotten Tomatoes, 7.9 IMDb) and *Logan* (94% Rotten Tomatoes, 8.1 IMDb), while others have been less well rated. In 2019, Disney acquired Fox and thus the rights to X-Men, ending an era of Fox X-Men movies and opening the possibility of incorporating X-Men into the MCU.

Unlike our world, the X-Men universe does not revolve around congressmen and people in power. Instead, characters like Senator Kelly are only foils or obstacles to the main characters who are always mutants. Mutants, in these movies, are people with a mutated X-gene that grants them special abilities. Their stories give us vivid, if fictional, portraits of how people live with an othered identity, and the various ways they react to the challenges of oppression. On a personal and social level, being genetically different puts a strain both on one’s psyche and on one’s familial ties. As portrayed in the world of X-Men, mutants often leave their biological families, searching out other mutants with whom they can form fulfilling relationships and perhaps create a new, “chosen family” (Scott and Fawaz, 2018; Blum, 2022). But beyond family structures, mutants in the X-Men films vary greatly in their ideas of what mutant-kind’s relation to society should be. Some, like Professor X, want to assimilate and aid the wider society, while others wish to strike first out of a sense of self-preservation, like Magneto.

While a mutated X-gene is the (scientifically implausible) foundation of mutant identity, there are frequently additional genetic factors at play in the films. Mutants’ amazing abilities lead many mutants and non-mutants alike to wonder about their humanity. Are mutants more than human? Or different enough to be another species? These questions are often raised explicitly, and the explanations given by the movies offer a glimpse into popular misconceptions about speciation, mutation, and human evolution. Additionally, since the basis of mutant identity is genetic difference, many of the dangers mutants face are linked to genetic conditions or biological traits. As they negotiate these issues, mutants run a gauntlet of scientific, medical, and bioethical transgressions: they are lied to by doctors, scientists, and government officials; they are turned into unwilling subjects in biomedical experiments; they are subjected to “cures” that they do not want; and their genetic data is stolen and then used in biowarfare against other mutants.

As a genetically different, othered population, mutants constantly face incomprehension, fear, hatred, and oppression from the wider society. In response, mutants form chosen families and participate in mutual support activities. The mutant stories shown in the movies are evocative of real-world oppression experienced by othered groups, including queer people, people with disabilities, racial minorities, and ethnic groups. But in their own universe, mutants’ difference comes from a mutated gene, and consequently, the films draw attention to (even as they distort) many scientific, medical, and bioethical concepts.

We argue that the X-Men movie franchise expands the mutant-as-other metaphor through a plurality of tropes, allowing viewers who identify with diverse racial, ethnic, class, ability, gender, and sexual orientations to see themselves reflected on screen. At the same time, the films’ conception of diversity is thin and imprecise. The films’ market-driven appeal to diverse audiences cannot escape the surrounding culture, shaped as it is by white supremacy. The result is a limited vocabulary for thinking about difference.

In the approximately 60 years since Marvel Comics introduced X-Men, the mutants have tended to be read through the lens of specific othered populations. The first series of X-Men comics debuted in the 1960s when the civil rights movement was in full swing. Hence it was no surprise that anti-mutant bigotry was often associated with prejudice against African Americans. The revival of X-Men in a new comic book series in the post-Stonewall era, together with political mobilization in response to the AIDS epidemic, led many readers in the 1980s and 1990s to associate the X-Men with LGBTQ+ rights. Public awareness of systemic problems such as racial injustice, transphobia, and homophobia has only grown in the twenty-first century, concurrent with the release of the X-Men films. Consequently, the versatility of the mutant metaphor has enabled the X-Men movies to become vehicles for social critique, an unusual position for summer blockbusters.^3^ This critique, however, comes at the cost of hollowing out the specificity of different minoritized populations in the service of a fluid metaphor of mutant-as-other. Genetics is what most powerfully facilitates this hollowing out of difference. When any kind of difference—physical, mental, social—can spring from a single source, the mutated X-gene, then all kinds of difference become versions of the same. The result is a panoply of wildly different beings who are paradoxically almost interchangeable as allegories for difference itself.

This paper examines the relationship between genetics and othering across the entire series of X-Men films. Section I outlines the background of the films in relation to the original Marvel comic book series and discusses the development of the theme of othering prior to the first cinematic versions. Section II reviews the extensive critical work on X-Men comics and films, as well as relevant aspects of the cultural discourse that surrounds these texts. Section III provides information about our method of collecting and analyzing the thirteen films. Section IV details the quantitative results of our investigation, while Section V turns to qualitative analysis to reveal how the relationship between genetics and otherness is depicted in representative scenes across the entire canon of X-Men films. This section is sub-divided into three principal topics: genetic difference, family, and otherness. Section VI is the conclusion.

### Background

This paper focuses on the thirteen X-Men movies that have been released over the last two decades, but these now-popular superheroes have a longer, more checkered history in the comic books where they originated. The group of mutants known as the X-Men premiered in a comic book written by Stan Lee and illustrated by Jack Kirby in 1963 (Hiatt, 2014). Marvel Comics had achieved great success in the creation of superheroes such as the Fantastic Four, Iron Man, and the Incredible Hulk, and was looking to introduce a new series. Having exhausted radioactive origin stories, Lee wanted to do something different with his newest creations. “I figured, hey, the easiest thing in the world: They were born that way. They were mutants!” (Hiatt, 2014). At that time the X-Men consisted of Professor X, their leader, and his five white American students, Cyclops, Jean Grey, Beast, Iceman, and Angel.

When the series first premiered, it was considered less than a success and was canceled in 1969 (Marvel Entertainment, 2019a). In 1975, however, in the context of the continued social advances of the civil rights and gay rights movements, writer Len Wein and artist Dave Cockrum were tasked with revamping the X-Men for a new era, beginning with a special issue titled *Giant-Size X-Men*. With the goal of making their new team as international and diverse as possible, Wein and Cockrum introduced now-iconic characters such as Nightcrawler, Storm, Colossus, and Wolverine who hailed from Germany, Egypt,^4^ Russia, and Canada respectively (Miller, 2003; Marvel Entertainment, 2019b). These characters have all appeared in the X-Men films alongside the classic X-Men members and laid the groundwork for continued diversity in the X-Men universe. While the increased diversity of the 1970s X-Men is notable in contrast to the initial team, it did not go much farther than gesturing toward different national identities.

Chris Claremont, who also began work on *Uncanny X-Men* in 1975, is credited with creating the most recognizable versions of the X-Men characters today and for honing the metaphor of what it means to be a mutant, at odds with society. Depicted as social outcasts throughout all their incarnations, the X-Men have always served as symbols of what it means to be feared and othered by society for traits over which they have no control (Diaz, 2019). This more diverse era of X-Men was able to better emphasize mutants’ identity as social outcasts by depicting many intersectional identities, such as mutant and Black, mutant and indigenous, mutant and queer, and mutant and disabled. But these intersections all lean heavily on the mutant part of identity, sometimes stereotyping the other aspect of a mutant’s character. Under Claremont, the X-Men became one of Marvel’s most popular comics.

Those who grew up in the 1990s or early 2000s may be more familiar with the X-Men characters from the popular animated TV series, *X-Men: The Animated Series* (1992–1997) and *X-Men: Evolution* (2000–2003). There was also another short-lived series that ran concurrently with the movies, X-Men Origins: Wolverine (2009) and a later live-action show, *The Gifted* (2017) (IMDb, 2021). The television shows and the movies have Xavier’s School for Gifted Children as their centerpiece, where young mutants go to learn in a space safe from the prejudices of the outside world. This sets X-Men apart from other superhero franchises because it allows for the inclusion of children and adolescents as both main and side characters. The movies are concerned with the lives and thoughts of young people, the target demographic of summer blockbusters, who are likely to see themselves represented on screen because of the diversity of characters.

While the X-Men TV shows and even early comic books can now be found online, they initially were aimed at U.S. audiences, with international readers and viewers always secondary. Mutants hail from all over the world, and while Xavier’s School for Gifted Children is certainly a factor in bringing mutants to the United States, specifically New England, it does not fully explain the U.S. centrism of the X-Men stories. The American focus of the X-Men might be less notable if it were not for the increased diversity within the X-Men that began in the Claremont era of the comic books and the high international box office revenues. With the premiere of the movies, increasingly diverse audiences were consuming X-Men content that either lacked in-depth portrayals of minoritized characters or provided imprecise or problematic storylines riddled with problems a white American audience might miss. All the same, the mutants of the X-Men remain popular because of the flexible core metaphor of genetic otherness.

### Review of criticism

The X-Men comics and films have attracted substantial scholarly and critical interest. Many academic critics have dedicated their efforts to the original medium of the Marvel comics, with topics of robust discussion including identity formation (Kellner, 1992; Zingsheim, 2011a; Zingsheim, 2011b; Lund, 2015), superheroes as reflecting the “American dream” (Trushell, 2004), issues of ethics (Gerde and Foster, 2008), the Holocaust and its trauma (Weinstein, 2006; Malcolm, 2010; Wenger, 2010; Royal, 2011; Smith, 2017), LGBTQ+ sexuality (Alexander, 2018; Doran, 2020; Bikowski, 2021), closetedness (Johnson, 2020), and issues of race and gender (Pierce, 2009; Nama, 2011; Darowski, 2014; Evans, 2019). In a special issue of *American Literature* titled “Queer about Comics,” editors Darieck Scott and Ramzi Fawaz emphasize what they see as the fundamental queerness of the medium of superhero comics (2018). The Introduction to the issue investigates the varied depictions of difference featured in X-Men comics, ranging from constructs of race to the establishment of queer kinship, but it locates these themes as a distinctive affordance of the comic book medium, not the films. In another contribution to the issue, Anthony Michael D’Agostino explores the multilayered depiction of difference presented in the character of Rogue. D’Agostino recognizes Rogue as invested with qualities of queerness that go beyond bids for tolerance and attempts at diversity by conceptualizing “the relationship between difference and consciousness and generat[ing] new possibilities for affiliation, solidarity, and recognition that have yet to reveal themselves in the ‘real world’” (D’Agostino, 2018). However, this complex psychic, social, and sexual positioning of Rogue is a feature of Claremont and Golden’s vision of the character in the comic books and does not come across in the sentimental and heteronormative longings of Rogue in the films.

In the public sphere, newspapers, magazines, and various digital media outlets have used Marvel comics to make connections between fantasy and reality, whether by singling-out problematic politicians (Advocate, 2006), spotlighting cultural and representative milestones of the franchise (Perpetua, 2012), recurring to the Civil Rights Movement (Ciampaglia, 2023), noting the presence of assimilationism (Blakemore, 2018), or highlighting issues of race and identity (Lyubansky, 2011; Demby, 2014). Additional scholarly attention has come from museum exhibitions in Dearborn, Philadelphia, Chicago, Seattle, and Berlin. While some of these exhibitions primarily provide basic introductions to the Marvel universe, others have highlighted LGBTQ+ representation for public commentary and engagement.

Critics who have focused on the *X-Men* film franchise have emphasized transmedia storytelling (Wucher et al., 2014), concepts of justice (Smathers, 2016), examples of Self-Othering (Million, 2006), Jewish subtext (Baron, 2003; Ebbrecht, 2010; Taylor, 2011), women and gender fluidity (Kent, 2021), the “trap of patriarchy” (Kaklamanidou, 2011), and references to German history and culture (Porter, 2021–22). While this list is by no means exhaustive of the many focal points scholars have singled out for cultural investigation, tropes of race and LGBTQ+ representation are notably under-researched in the scholarship on the X-Men films. This paper adds a focus on the role of genetics in shaping the presentation of difference, particularly in regards to race and LGBTQ+ issues. Christopher B. Zeichmann, for instance, explores how the first two installments of the X-Men films—*X-Men*, *X2: X-Men United*—utilize queer metaphors that he reads as “problematic to the extent that [they reinforce] historical and prevailing real-world anti black respectability politics” (Zeichmann, 2020). He argues how the first two installments to the film franchise “are emphatically anti-intersectional, in that queer liberation and Black liberation are placed in an antagonistic and ultimately unreconcilable relationship, wherein the former is lent legitimacy by its refusal of violence and insistence upon gaining inclusion within hegemonic social structures that perpetuate the marginalization of people of color” (Zeichmann, 2020, 392). As the franchise has progressed, it has evolved to address more intersectional topics pertaining to systemic oppression and subjugation of minoritized groups, especially as they relate to issues of genetic privacy and the exploitation of biological materials by both commercial and governmental entities.

**FIGURE 1:**
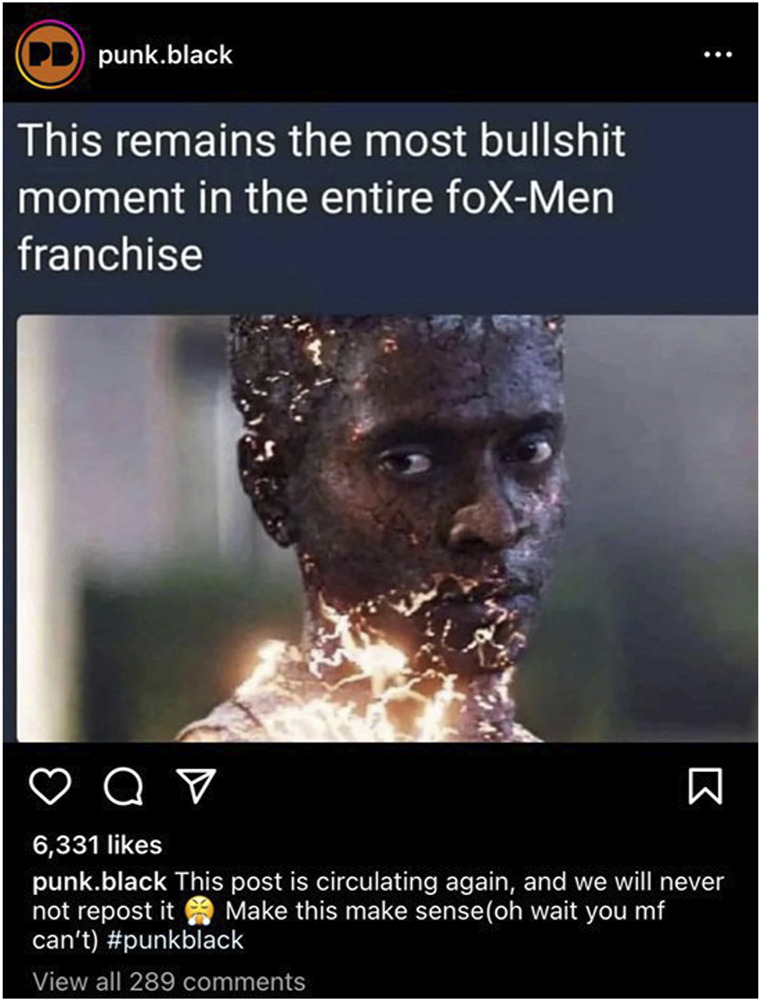
@punk.black, Instagram. 30 August 2023. X-Men: First Class, (2011), Darwin succumbing to Shaw’s attack, despite his powers of indestructability. [Accessed 24 October 2023].

Social media has served as an additional sector where subjects of race, ethnicity, class, and LGBTQ+ representation are actively discussed, often in an intersectional context. Platforms like Instagram, Reddit, Twitter, and Fandom.com serve as communal spaces where X-Men buffs meet to discuss, chastise, scrutinize, and air out frustrations tied to decisions Marvel has made in the presentation of its characters and the unfolding of X-Men cinematic narratives. Take, for example, the demise of the X-Men character Darwin in X-Men: First Class (2011). The circumstances of his death reminded American audiences of the conventions that plague Black characters on the big screen, regardless of their superpowers and resilience. Darwin is a mutant with “the power of ‘reactive evolution.'” His body automatically adapts to any situation or environment he is placed in, allowing him to survive almost anything; the exact nature and limits of his powers have not yet been revealed” (X-Men Wiki, 2023). The circumstances of Darwin’s (Edi Gathegi) demise in *First Class* are reminiscent of the sacrifice Black characters are often called on to make in Hollywood films. In an effort to protect his white peers and his notably fair-skinned and hegemonically beautiful Black female counterpart, Angel Salvadore (Zoë Kravitz), Darwin is killed. While the Darwin of the comic universe could withstand anything and everything thrown his way, needing only to put his instantaneous adaptive powers to work, the cinematic rendition of Darwin fails to survive the encounter, becoming an example of the cinematic trope Coleman and Harris draw attention to in the title of their monograph, *The Black Guy Dies First: Black Horror Cinema from Fodder to Oscar* (Coleman and Harris, 2023)*.* This persistent failing of Hollywood cinema has not gone unnoticed by the series’ fanbase, as witnessed by the angry meme in Figure 1.

## Methods

To analyze motifs of genetics in relation to othering in the X-Men film franchise, we first identified the relevant canon and then acquired transcripts of each film. The thirteen X-Men movies released by Fox were cataloged according to year and linked to a brief identifier (X#). The transcript for each movie was collected from a British fan site that makes available a wide range of movie and television show scripts (Springfield). These transcripts were copied into a txt file, where each line of dialogue could be numbered individually. Table 1 shows the film order, titles, years released, and directors.

**TABLE 1:** Movie list.

X#	Title	Year	Director
X1	X-Men	2000	Bryan Singer
X2	X2: X-Men United	2003	Bryan Singer
X3	X-Men: The Last Stand	2006	Brett Ratner
X4	X-Men Origins: Wolverine	2009	Gavin Hood
X5	X-Men: First Class	2011	Matthew Vaughn
X6	The Wolverine	2013	James Mangold
X7	X-Men: Days of Future Past	2014	Bryan Singer
X8	Deadpool	2016	Tim Miller
X9	X-Men: Apocalypse	2016	Bryan Singer
X10	Logan	2017	James Mangold
X11	Deadpool 2	2018	David Leitch
X12	X-Men: Dark Phoenix	2019	Simon Kinberg
X13	The New Mutants	2020	Josh Boone

Each movie was watched twice with the closed captions on to correct the transcript to include only spoken dialogue that audiences would hear. The first viewing of the movie was used to correct words, punctuation, and formatting. The second viewing served to double-check the transcript, eliminate non-meaningful sounds (uh, bleep, achoo), and languages other than English (with the exception of clearly intelligible loan words such as “nein,” “sayonara,” etc.). In this stage, we attended to formatting details of the transcript txt files, such as inserting a new line every time there was a new speaker.

After collecting and cleaning the transcripts, we determined our unit of analysis. Considering each movie in its entirety would not yield the nuanced discriminations of attitudes toward genetics and otherness that we were seeking. On the other hand, examining every line of dialogue in thirteen films would be unwieldy. Consequently, we settled on using the scene as our unit of analysis. But what constituted a scene? Different media have different ways of telling stories, including their internal organization. A play in the theatre may have relatively few scenes, which may be clearly demarcated by set changes. Movies, particularly summer blockbusters that feature frenetic action sequences like the X-Men films, cut frequently from character to character and place to place. Scene divisions are not always clear and obvious.

After discussing the nature of a scene and camera cuts, we agreed upon the following general rule: if the action occurred in a new place, new time, or involved new characters, it constituted a new scene. But even then, distinguishing tiny scene shifts within a larger dramatic arc distracted from the focal themes, so we grouped smaller or overlapping scenes together as one sequence. This methodological choice was particularly useful in linking the quick cuts between combatants in fight scenes together as one, extended fight sequence. As with many other blockbuster action movies, the fight scenes in X-Men movies often display entertaining and creative choreography but not a lot of meaningful dialogue, thus diminishing their pertinence to the movie’s themes of genetics and othering.

Key details were documented for every scene, including the timestamp of when the scene began, the numbered transcript lines spoken, the setting, a brief description of the action, a list of the characters present, and additional notes. Once all the movies had been demarcated by scene, new txt files for each scene were created using a python script. All the scenes were then uploaded to MAXQDA, a qualitative coding software. The runtime, transcript word count, and number of scenes for each movie are presented in Table 2.

**TABLE 2:** Movie data.

X#	Title	Runtime (minutes)	Word Count (transcript)	Total Number of Scenes	Scenes with Dialogue
X1	X-Men	104	4,835	52	46
X2	X2: X-Men United	134	6,958	53	49
X3	X-Men: The Last Stand	104	5,819	57	54
X4	X-Men Origins: Wolverine	107	5,537	50	46
X5	X-Men: First Class	131	9,359	61	60
X6	The Wolverine	126	5,000	43	41
X7	X-Men: Days of Future Past	132	8,052	40	37
X8	Deadpool	108	8,751	42	40
X9	X-Men: Apocalypse	144	7,838	57	55
X10	Logan	137	7,882	55	52
X11	Deadpool 2	119	10,913	44	41
X12	X-Men: Dark Phoenix	113	6,397	43	43
X13	The New Mutants	94	5,245	47	43

We developed a codebook to document themes across the films (see Supplementary Material for the complete codebook). We began by discussing our two thematic foci, genetics and othering. With regard to genetics, we identified genetic topics that we knew arose in the X-Men films from previous viewings, such as human evolution, the fictious X-gene, the so-called “cure” for mutation, and more. These codes were grouped under one of our code categories, genetic themes. For othering, we identified motifs associated with racialized populations and queer people. These led to the code categories of *Race* and *LGBTQ+*. We also wanted to capture how mutants viewed themselves (*mutant attitudes and affects*) and how society at large viewed mutants (*society’s view/treatment of mutants*). We created a code category for *character demographics* to note whether scenes included only mutants or non-mutants. Finally, we coded for *social factors*, which covered other relevant themes like family, adolescence, gender, religion, and more.

To determine the codes within each category, we used an inductive approach. Starting from an initial broad discussion of themes, we continually added to our list of themes as we identified them in our viewing of the movies. For example, in X-Men: First Class (2011) there is a scene in which a character is “outed” as a mutant, which led us to create the code “outing.” After discussion, we also added the code “closet” to refer to a closeted mutant, since there could be scenes involving a closeted mutant who is not outed. Our coding continued in this way until all relevant themes had been captured in the codebook, a total of 99 codes.

Once we had our codebook, the authors watched the movies again and coded them in MAXQDA. A code could be identified with an entire scene, or with a specific section of dialogue. If a theme was signaled visually but not verbally, the coder would mark the adjacent dialogue with the code and write a memo in MAXQDA. Early in the coding process, three of the authors coded the same movie separately, then met to discuss and consolidate our codes to elevate inter-coder reliability. The movies we knew from our first viewings to be the richest in themes were double or triple coded (X1, X2, X3, X5, X7, X9, X10, X11, and X13), while the movies with less thematic content were coded only once (X4, X6, X8, and X12).

## Quantitative results

In total, across the thirteen movies, 3,459 coded segments were identified by the research team. The seven code categories and their aggregated code totals are listed in descending order in Table 3. The bolded values are the total number of qualitative codes for that category. For a complete list of codes, see Supplementary Material.

**TABLE 3:** Primary code categories.

Code category	Number of subcodes	Total
Social factors	25	1,142
Mutant attitudes and affects	13	728
Society’s view/treatment of mutants	14	539
Genetic themes	22	348
LGBTQ+	10	296
Character demographics	4	262
Race	8	144
	99	3,459

*Social factors* is the largest category, making up a third of all codes. This is not surprising as it has the most codes and covers everyday social themes that appear frequently in the X-Men world. When the subcodes are combined into their parent code totals, social factors contain the top two most frequent codes, *family* (*n* = 251) and *state institutions* (*n* = 142), which are both highly relevant to mutants’ othered experience. The subcodes for family are *biological family* (*n* = 121) and *chosen family* (*n* = 110). As we will explore in the analysis section, mutants’ genetic difference potentially isolates them from their biological family, and in their isolation, they often turn to other mutants for connection and form chosen families. State institutions, such as the *legislative branch* (*n* = 10), the *military* (*n* = 78), which is part of the executive branch in the United States, and other state actors (*n* = 54) make up the governmental systems that oppress mutants by reinforcing their genetic difference. These institutions reinforce mutant difference by enacting harmful policies against them, like the mutant registration bill previewed in one of the epigraphs of this paper.

The next three most frequent codes all come from *mutant attitudes and affects* and share similar totals, *anger* (*n* = 121), *mutants*’ *fear* of themselves or others (*n* = 120), and *emotional stress* (*n* = 118), which includes emotions such as anxiety, despair, grief, and pain. Besides the mutants-only demographic code, these codes most commonly occur with each other, particularly emotional stress which is the primary co-occurrent code for mutants’ fear (29%) and anger (22%). This is indicative of the main focus of X-Men movies, telling mutant stories. Mutants’ lives involve a great deal of emotional turmoil as they face the challenges of living with an othered identity.

Mutants’ otherness is demonstrated in two codes that frequently co-occur, discrimination against mutants that is racially coded and discrimination against mutants that is LGBTQ+ coded. *Discrimination against* (*LGBTQ+*) is the second highest occurring code in the *LGBTQ+* section at *n* = 35 coded segments (Table 4). *Discrimination against* (*race*) is the most frequent code in the larger *Race* category, at *n* = 41 coded segments (Table 5). These codes overlap in 17 segments, such as when Mr. Worthington says, “I only wanted to help you people” (X3, Scene 56). “You people” could be a reference to mutants, African Americans, queer people, Jewish people, or another othered group, depending on the context.

**TABLE 4:** LGBTQ+ codes.

		Total
LGBTQ+		10
Passing		52
Discrimination		35
Isolation		34
Cure		34
Closet		32
Name		30
Outing		29
Difference		22
Queer relationships		13
Homophobia		5
		**296**

**TABLE 5:** Race codes.

		Total
Race		16
Discrimination		41
Excessive force		29
Revolution		15
Underground railroad		13
Separatism		12
Slavery		8
Harassment		8
White supremacy		2
		**144**

Society’s view and treatment of mutants is overwhelmingly negative in the X-Men movies, as seen in Table 6. The most frequent code in this category is *fear* of mutants (*n* = 92) and the third most frequent code is a generalized *hostility* towards mutants (*n* = 74). The passages coded for fear or hostility rarely have engaging dialogue or an explanation for why someone is afraid or hostile towards mutants. Fear is not rational, though many mutant antagonists justify their hostility by claiming that mutants are inherently violent (*n* = 60) and dangerous (*n* = 50). This rhetoric evokes the long-standing white myth that Black people are exceptionally violent. One of the consequences of that myth in our own world—high rates of African American incarceration—can be seen in the X-Men universe as well, with *imprisonment* (*n* = 85) as the second most frequent code for how society treats mutants. Not all responses to mutants are negative, as can be seen with the two positive codes in this category, *allies* (*n* = 31) and *admiration* (*n* = 17), but their totals are substantially lower than the unfavorable codes.

**TABLE 6:** Society’s view/treatment of mutants codes.

Society’s view/treatment of mutants	Total
Fear (non-mutants’)	92
Imprisonment	85
Hostility	74
Violence	60
Dangerous	50
Warfare	44
Allies	31
Surveillance	28
Oppression	27
Admiration	17
Us (humans) against Them	14
Conspiracy	10
Mind control	4
Secrecy	3
	**539**

Although genetic difference is the core of mutant identity, some genetic motifs appear more frequently than others (Table 7). Out of the 21 codes in the category of *genetic themes*, only 9 codes have a total greater than 10. Motifs such as cloning, genealogy, genetic engineering, and radiation appear in the films but only sporadically. The most prominent genetic themes documented are *experimentation* (*n* = 64) and *mutation* (*n* = 55). Mutation is to be expected since these are movies about “mutants,” but the prominence of experimentation is noteworthy. Experimentation, especially in racial and eugenic contexts, are explored in the qualitative analysis.

**TABLE 7:** Genetic themes codes.

Genetic themes	Total
Experimentation	64
Mutation	55
Science talk	33
Longevity	30
Evolution	26
Extinction	25
Posthumanism	16
Speciation	16
Biological Warfare/Bioterrorism	14
Genetic condition	9
Radiation	9
X-gene	8
Genetic engineering	7
Genealogy	6
Bio-banks	4
Eugenics	4
Cloning	3
Nature v. nurture	3
Genetic screening	2
Forensics	1
Deep time	0
	**335**

The most prominent codes across the corpus of X-Men movies reveal that despite their occasional niche genetic content, relatability is at the core of these stories. *Family* is by far the largest code, with 109 more coded segments than the second highest code, *state institutions*. Dealing with family is a timeless experience that all audiences can relate to; interacting with state institutions like the government or military is also commonplace in our modern age. Feelings like anger, fear, anxiety, despair, grief, and pain bond viewers to the mutants on screen, as surely everyone can recall feeling such emotions at some point in the past. While *Race* and *LGBTQ+* offer the two most obvious parallels for mutants, the prominence of the *discrimination* code in both categories demonstrates that the mutant metaphor is not limited to either allegory. The expansiveness of mutants is perhaps best shown through their opponents, who express general *fear* and *hostility* towards mutants and act in ways that harm them. The ensemble nature of these movies allows for a wide variety of character arcs and identity intersections, while remaining relatable to a wide audience.

## Qualitative analysis

### Genetic difference

In the world of the X-Men, mutants are people who possess the (fictitious) X-gene. Their mutations grant them a variety of special abilities, including flight, teleportation, telepathy, telekinesis, bodily transformation, control over elements, and more. Anyone with some genetic literacy would realize that it is impossible for mutations in a single gene to produce such superhuman powers, much less an entire array of superpowers, but ludicrous science is not our concern in this paper. Instead, we focus on the allegorical and thematic implications that arise from the notion of genetic difference. Whether it is an unidentified mutation or the X-gene in particular, mutants are perceived as different from the majority because of their genetic code. Perhaps the most obvious indicator that their otherness arises from their genetics is the word used to describe them: mutants.

Because of their difference, both mutants and non-mutants alike wonder where mutants fit in on the human family tree. Many characters explore mutant identity through ideas of evolution and speciation. Professor X (Patrick Stewart) wonders out loud, “are mutants the next link in the evolutionary chain?” (X2, Scene 1), while Magneto (portrayed in some movies by Michael Fassbender) confidently states, “We’re the next stage of human evolution” (X5, Scene 57). Passages such as these, and there are many, reveal an underlying assumption that evolution is linear, moving from one “stage” or “chain” to the next. This popular misconception is famously illustrated in the metaphor of the March of Progress, which implies the false notion that a species cleanly evolves into another species that is “better” than the first.

Buying into the notion of a superior species, Magneto (also portrayed in some movies by Ian McKellen) promotes mutant superiority over non-mutant humans. Speaking to the United States President and the public via a television broadcast, Magneto proclaims, “You are right to fear us. We are the future. We are the ones who will inherit this Earth” (X7, Scene 37). Magneto believes that mutants are worthy of fear because of their powers, and since they are the “better” species, they “will inherit this Earth.” His response to being othered by the dominant group is to flip the script and declare mutants superior to humans. To counter the threat of a pogrom against mutants, Magneto sets out to destroy humanity instead and bring about a mutant-only future.

Professor Charles Xavier, or Professor X as he is most often called, opposes Magneto’s destructive plans, but he shares some of the same underlying views of evolution and speciation. Professor X was once a professor of genetics and X-Men: First Class (2011) depicts a young Charles Xavier (James McAvoy) as a PhD student at Oxford. Sometimes when his foster sister, later to be known as Raven (Jennifer Lawrence), was unable to fall asleep, Charles would read her part of his thesis on Neanderthals, our human cousins who went extinct around forty thousand years ago.

Charles: To *Homo neanderthalensis*, his mutant cousin, *Homo sapiens*, was an aberration. Peaceful cohabitation, if ever it existed, was short-lived… The arrival of the mutated human species in any region was followed by the immediate extinction of their less evolved kin.

(X5, Scene 6)

Charles emphasizes inter-species conflict, claiming that it is inevitable, and necessarily leads to “the immediate extinction” of the less-evolved species. His analysis of Neanderthal-Homo sapiens relations is not supported by modern human evolutionary scientists, who have shown that *homo sapiens* and *homo neanderthalensis* interbred and thus overlapped in time rather than there being an immediate eradication of the “lesser” species (Wolf and Akey, 2018; Villanea and Schraiber, 2019). Modern research has also revealed a complex web of factors that led to Neanderthal extinction, including conflict but also climate change (Timmermann, 2020). Though Charles’ thesis is inaccurate according to current scientific understanding, in the movies the belief that Neanderthals were driven to extinction by a newer, more evolved species (i.e., *homo sapiens*), serves to legitimate humanity’s fear of being eradicated in turn by a new species of mutants.

The notions of a superior species and an inferior species directly evoke the eugenics movement of the late-nineteenth and early twentieth centuries in the United States and Nazi Germany. In her article “Germans and Genes on Screen; Marvel’s *X-Men* Films,” Cynthia D. Porter investigates the many references to German history and culture found in the first three installments of the X-Men film franchise (2021–22). Porter’s analysis includes the interwoven histories of medical experimentation and national socialistic eugenic ideology rooted in the perceived superiority of Aryan genes. Porter writes, “Racial hygienists developed arguments based on perceptions of genetic strength, stating that genetic weakness was the determining factor in the identification and condemnation of targeted communities” (2021–22). She continues by presenting the many examples of forced or coerced experimentation on X-Men favorites—like Wolverine, Magneto, and Professor X—as representing what she argues is an invitation for the viewer to recall “the Nazi crimes against humanity, particularly pertaining to the issues of genetic privacy and bioethics” (Porter, 2021). Later installments of the X-Men film franchise continue the tradition of referencing the Holocaust, but with varying focal points and embedded critiques. Take, for example, villain Sebastian Shaw as he endeavors to reassure a young Magneto of their common interests by emphasizing what he identifies as the true value located in mutant bloodlines: genetic evolution.

Sebastian Shaw: These Nazis, I’m not like them. Genes are the key, yes? But their goals? Blue eyes? Blonde Hair? Pathetic.

(X5, Scene 3)

In the X-Men films, mutants are not known to the general public until 1973, and their perceived “newness” as a species contributes to a sense of the freakish Other. In *X-Men: Apocalypse* (2016), Alex, a member of the X-Men, says to CIA Agent Moira MacTaggart, “I thought mutants did not evolve until this century,” and she replies, “That’s the common theory, yes” (X9, Scene 16). In the movie, the common theory is proven false by the existence of an ancient mutant, but that does not change the widespread misconception that mutants suddenly appeared out of nowhere. The newness of mutants echoes moral panics over an “increase” in queer people which failed to recognize that same-gender relationships have existed in the past (Boswell, 1981).

As mutants emerge from the closet in the movies, they find a society that is not ready to welcome them. Mutants in the X-Men films experience a wide range of hostility and backlash rooted in fear, discomfort, and hatred. Perhaps one of their most notorious villains is Dr. Bolivar Trask (Peter Dinklage), the CEO of Trask Industries, who creates giant killer robots called the Sentinels in X-Men: Days of Future Past (2014). His aim is the total extinction of mutants and anyone who supports them, a complete genocide. Trask uses the rhetoric of speciation, conflict, and extinction when he says his goal is one that “could unite us [*homo sapiens*] as a species” (X7, Scene 27). To Trask and other characters like him, mutants’ genetic difference is intolerable, and their very existence is a threat to humanity.

In this hostile world, mutants’ genetic difference puts them at greater risk for abuse, including violations of their genetic privacy and autonomy in medical and scientific settings. Unethical experimentation on mutants is a threat that looms large in the X-Men universe, from the mutation-inducing ray of the first movie, X-Men (2000), to the most recent movie, The New Mutants, (2020), where five mutant teenagers are under constant bio-scanning surveillance. The most visible victim of nefarious experimentation is James Logan Howlett, known as Wolverine (Hugh Jackman), who appears in seven of the thirteen films. Wolverine’s mutation has given him bone claws that can protrude through his knuckles and an endless, near-instantaneous healing ability. His mutant talents are prized by Colonel Stryker, a military scientist with an anti-mutant agenda, who authorizes horrific experiments on Logan.

In a state of distress after Stryker ordered the murder of his girlfriend, Logan agrees to Stryker’s experiment in which an indestructible (and imaginary) metal alloy called adamantium is bonded to his skeleton. The military scientists failed to inform him beforehand of the intense pain he would experience in the procedure or its awful aftereffects. As in the notorious US Public Health Service Untreated Syphilis Study on African American males at Tuskegee, the medical procedures performed on Wolverine were not done for his benefit. Instead, they were undertaken to transform Wolverine into a super weapon for Stryker’s own purposes. In both cases, the perpetrators of the experiment viewed their subjects as less than human, not worthy of proper treatment and respect. While Wolverine technically consented to the experiment, he did so under duress, and like the men of the Tuskegee experiments, he was never informed of the long-term sequelae.

Years later, in Logan (2017), Wolverine is close to death because of the slow poisoning from the metal inside him, an echo of the men in the Tuskegee experiment who later died of syphilis. Mercenaries trying to capture a mysterious eleven-year-old girl intrude on Logan’s life. Professor X informs him that the young girl “[is] your daughter, Logan. Alkali has your genetic code” (X10, Scene 21). Unknown to Logan, a biomedical corporation known as Alkali Transigen had kept his DNA and created a child without his consent, utterly violating his privacy and autonomy. The company later injected his “daughter” Laura with adamantium, so she was being slowly poisoned in the same manner as her father. This multi-generational misuse of medical research is hardly unprecedented in the annals of American science, particularly where Black people are concerned. For example, Henrietta Lacks’s descendants were encouraged for years to come in for further blood draws under the guise of cancer screening, when in fact the aim was to collect biological material for research unconnected to their healthcare (Skloot, 2010).

There are other racialized elements in the treatment of Logan and his “daughter”, Laura. Colonel Stryker constantly calls Wolverine “an animal,” which on the surface is a reference to his name but is racially coded, since similar language has long used to describe people of color. Laura is also coded as “an animal.” She does not speak until the last part of Logan (2017), communicating through inarticulate noises and body language. Her captors treat her as a dangerous beast, evoking the false notion that Black people are more violent than whites. Stryker’s mercenary views Laura as property, asserting that the nurse who freed her “took something of mine when I was not looking.” (X10, Scene 5) The “something” in question was not a valuable object but a human being, The mercenary pursues Laura as she flees toward Canada evoking antebellum slavecatchers who pursued runaway slaves traveling north on the underground railroad. While Laura’s story is evocative of formerly enslaved African Americans, she is not Black and is rather coded as Hispanic since she is Spanish-speaking, resulting in a muddled metaphor of racialization.

Genetic difference is the core of mutant identity for people in the X-Men cinematic universe. Characters on both sides of the conflict, including Professor X, Magneto, and Dr. Trask, view mutants as a different race or species. These characters—and many others in the films—create a dichotomy between mutants and non-mutants, and assume that conflict between the two groups is inevitable. In different scenarios, mutants are reminiscent of queer people in the second half of the twentieth century, Black people who have been systematically failed by institutions including healthcare and medical research, and the European Jews who were killed in the Holocaust because the Nazis viewed them as inferior. One cannot identify a single real-world allegory for mutants because mutant identity intersects with every part of a person, just as our own gender, sexuality, race, ethnic background, neurodiversity, faith tradition, and more weave together within us. In the world of X-Men, “mutant status” is an exceptionally flexible category.

“Mutant status” can be such a flexible category in the X-Men universe because the mutant metaphor is built on a plurality of tropes about otherness. Since genetic difference is the basis of mutant identity, a plethora of themes associated with genetics emerge from this versatile metaphor, ranging from evolution, speciation, and extinction to eugenics and unethical experimentation on unwilling subjects.

### Family

The Deadpool films are among the few in the X-Men series that are rated R, and rightfully so, as they are filled with profane humor and violent, gory imagery. Hence it is a bit surprising when at the beginning of Deadpool 2 (2018), Deadpool announces “believe it or not, *Deadpool 2* is a family film” (X11, Scene 6). The term “family film” usually refers to a film that an entire family can enjoy, including young children, but what Deadpool means in this case is that the intensely violent film that follows ends up being a story about a family. We never meet or learn about Wade Wilson’s parents or siblings, but by the movie’s close, it is clear he has acquired a family, one forged out of elective affiliation rather than biological kinship.

The phrases “chosen family” or “found family” refer to familial ties that do not have a biological basis. Membership in a chosen family is not based on sharing a recent common ancestor, but rather on choice; everyone has chosen to commit to one another. Because it is disruptive to the traditional heteronormative ideal of the nuclear family, found families are inherently queer family structures (Weston, 1991; Blum, 2022). But the queerness associated with chosen family is also related to the higher likelihood that queer people will be in a found family because they have been rejected by their biological family.

Mutants can face rejection or alienation from their biological family when their mutation reveals itself around puberty. As a doctor says in The New Mutants (2020), “Mutation most often occurs in puberty. You might spend the first 13 years of your life relatively normal. Then… you come of age and discover your true nature” (X13, Scene 3). This scenario is utterly wrong scientifically, but it chimes with the experience of some LGBTQ+ youths, whose discovery of their sexuality can be disruptive to their sense of self, and their familial and social ties. In *X-Men* (2000), after her mutation manifests, Rogue leaves her family in Louisiana, journeying all the way to Canada by herself. In The New Mutants (2020), five mutant teenagers are trapped in a mysterious clinic, separated from their homes and families. In X-Men: Dark Phoenix (2019), after the death of her mother, Jean Grey is relinquished to Professor X by her father who no longer wants to be responsible for her. And in Deadpool 2 (2018), Russell is a teenager being abused at a mutant orphanage, with no supportive adults in his life. Teenagers facing hardships, often of the familial variety, remain at the center of X-Men stories.

Professor X: I’ve come to bring you home.

Jean Grey: I have no home.

Professor X: Yes, you do. You have a home and a family [now].

(X3, Scene 32)

As with other genetic traits, the fictional X-gene is not necessarily expressed in every member of a family. It is completely possible for one child to be a mutant and have non-mutant parents and siblings. The emergence of mutation at puberty combined with the recessive nature of the X-gene contributes to the mutants-as-queer analogy, since queer sexuality often becomes apparent at puberty and sexuality can vary among siblings. The mutant family tropes seen throughout the X-Men movies are almost direct retellings of common LGBTQ+ coming out stories, with superpowers standing in place of gender or sexuality. The most obvious example is in X-Men Apocalypse (2016), when Bobby Drake returns home with his friends from mutant school and must “come out” to his family. His parents’ and brother’s reactions are not positive; his mom even asks, “Have you tried not being a mutant?” (X2, Scene 38). But a snarky comment from Bobby’s classmate is a reminder that even if his family chooses to distance themselves from him, their genetic connection remains.

Bobby’s mom: This is all my fault.

Pyro: Actually, they discovered that males are the ones who carry the mutant gene and pass it on, so it is his fault [gesturing to Bobby’s father].

(X2, Scene 36)

The queer overtones of this coming out scene and Pyro’s reference to “the mutant gene” and “his fault” evoke genetic essentialism and the concept of the gay gene, which is still debated as scientists and lay people are unsure about the extent to which queerness is genetic (Hammack-Aviran et al., 2022). Pyro’s bitter rejoinder also plays on the notion that difference is someone’s “fault,” but the scene makes it abundantly clear that we are not meant to interpret this otherness as bad—quite the reverse. Rogue, Bobby’s girlfriend, tries to articulate that point, insisting to his mom, “Bobby *is* gifted.” Bobby’s parents remain hesitant and uncomfortable in the presence of their mutant son and his mutant friends. Bobby’s brother goes further in his animosity, leaving the room to secretly call the police.

Bobby’s “coming out” scene is further complicated by his brother’s phone call to the police, which swiftly shifts the tone of the family visit from one connoting queerness to one connotating Blackness. Mistreatment of Black adults, particularly men, by law enforcement has been amply documented in recent years (Desilver et al., 2020). When the police arrive, the scene plays out in a scenario familiar from countless incidents of police violence toward Black people. Logan is attempting to deescalate the scene by explaining that the call was all a misunderstanding, but when Logan’s “knives” inadvertently emerge, the police open fire. Even though Logan has raised his hands and is not threatening anyone, law enforcement overreacts, endangering innocent bystanders with a hail of gunfire. Making the scene even more troubling is that it endangers children. The officer’s patronizing attitude toward Pyro, calling him a “kid” but treating him as an adult, evokes the dangerous and detrimental American history of “adultrification” of Black children (Koch and Kozhumam, 2022; CPE Staff, 2023). A 2014 American Psychological Association study of police behavior towards young Black boys found that “dehumanization and not police officers’ prejudice against Blacks—conscious or not—was linked to violent encounters with Black children in custody” (APA, 2014; Goff et al., 2014). In this scene, the police dehumanize mutants in the same way, viewing them as an inhuman threat to contain rather than acting out of conscious hatred. However, the analogy to Black experiences of police violence is incomplete since all the mutants in this scene are White.

Even if a mutant “coming out” goes relatively well and does not end in a police confrontation, there can still be danger ahead for mutants and their families, depending on the specific mutation a person has. Mutants like Jean Grey and Professor X have telepathic abilities, they can read minds, but their neurodiversity can also lead to heightened stress and confusion. Jean and Professor X experience sensory overload, like many people on the autism spectrum, and their minds and thinking processes are often misunderstood by their fellow mutants. Some mutants face the different problem of longevity; they have an increased lifespan, usually due to a limitless healing ability. Wolverine, Sabretooth, Apocalypse, and Mystique all have longer than average lifespans and continue to look relatively young throughout the decades. The longer someone is alive, the fewer direct biological kin they have, unless a family member has a similar mutation (for example, Wolverine and Sabretooth are brothers). Mutants with longevity tend to create chosen family connections since their biological family may be long gone. Wolverine often takes a young mutant under his wing becoming something of a father figure to characters like Rogue, Yukio, and the unknown girl who turns out to be his actual daughter, Laura. A chosen family is more flexible than a nuclear family, and relationships can be more fluid and less defined. Although Wolverine may play a father-like role in her life, he is adamant that he is not Rogue’s actual father.

Rogue: Should not you be telling me to stay? To go upstairs and unpack?

Wolverine: I’m not your father. I’m your friend.

(X3, Scene 34)

Deep friendship combined with a shared genetic identity often brings mutant characters together in fulfilling relationships. Although they frequently are adversaries, Professor X and Magneto refer to each other as “old friend” across multiple films. They grow apart over time, but they never forget that they were once young men with a shared dream of making the world a better place for mutants.

Mutants in the X-Men films might experience conflict and rejection from their biological families, but they are often able to find and forge new families with other mutants. A mutant teenager may have to “come out” to their family, as queer teens often do, and they run the risk of rejection and alienation. Within or connected to the trope of queerness are behaviors and attitudes towards mutants that can be interpreted as potentially addressing a range of minoritized groups. Summarizing the application of the “mutant metaphor” in the X-Men comics, Joseph J. Darowski states that the “series does clearly and frequently use the concept of ‘mutants’ to explore issues of prejudice” (2014, 155). Bobby’s coming out scene is a locus from which the movie is able to explore both anti-queer and anti-Black prejudice, the latter in a violent conflict with the police. Fortunately, even in the face of prejudice, the mutants in X-Men films are able to make life-sustaining friendships that may evolve into a familial bond, premised not on direct kinship but on a shared otherness.

### To be othered

Though the movies give a sense of the broader world the X-Men live in, the films are primarily focused on a diverse ensemble of mutants. X-Men fans love to talk about their favorite characters because there are so many to choose from, each with a unique background, personality, and powerset. The individuality and humanity that fans love contrasts with the attitude of the mutants’ antagonists, who seem to only see a unified group of dangerous creatures, the fear-inducing “Other.” In fact, mutants are not a unified front in any sense; they vary in their self-perceptions, self-expressions, and ideals for mutantkind.

Mutants’ self-conception is often connected with the relative visibility of their “difference.” Some mutants are indistinguishable from non-mutants; their powers do not affect their appearance (Professor X, Magneto, Storm, Jean Grey). For others, their mutation is visually distinct, as in the cases of Mystique, Beast, and Nightcrawler, who share the trait of having blue skin. Others represent a composition of anonymity coupled with easily identifiable difference, as in the case of Angel, who has giant wings that are only hidden with great difficulty. Additional examples include Cyclops, who must wear special glasses to stop his laser eye beams, Wolverine, whose adamantium claws are retractable but terribly threatening when out, or Rogue, who appears “normal” but is incapable of skin-to-skin contact with another person, lest she drain their life-force. Even when members of the latter group are not using their powers, they remain easily identifiable as mutants. They cannot “pass” as human all of the time.

Kat Overland notes that the term *passing* “is most often applied to discuss race, but can be used more broadly to cover sexual orientation, ethnicity, or gender identity as well. Passing means that someone can lose an assigned status as the Other, gaining privilege and social acceptance by being perceived as someone they are not” (2009, 196). Placing the historical context of passing in conversation with its presentation in the X-Men films, Overland writes

In the United States, racial categories historically have been regulated in part to prevent passing—the “one-drop rule” meant that someone could not ascend from the status of a slave simply by looking “white enough” under the eyes of the law. Transgender people have plenty of reason to consider passing—violence against trans individuals is statistically much higher than for others, and there is still social and legal stigma against them (Giovanniello 2013) …. The conceptual connection between these examples and the X-universe is notable. Throughout the series, mutants are plagued by government entities looking to lock them into a subordinate class—from the Mutant Registration Act of the first film to the Sentinels program of *Days of Future Past*, the law is a tool with which to contain mutants by codifying their social identity as forever and Other (Overland, 197-98).

The subject of passing is at times presented in the X-verse as a means of protection—but one that is perceived by the human antagonists of the films as inherently threatening. The groups of mutants that can go undetected are understood by government officials as a domestic threat. The threat arises from the possibility that a human could work with or be friends “with a mutant without ever being the wiser. Passing creates the possibility that someone’s humanity could be ‘performed,’ that it could be constructed or worn as an identity rather than being an innate characteristic” (Overland, 2016, p. 200). A consistent theme in the X-Men films, governmental intervention with the goal of preventing true integration of minoritized groups has historically been focused on maintaining the hierarchical *status quo*. Overland continues by explaining that “[p]assing disrupts what was once an intransgressable line, so the status conferred to white[ness] becomes less exclusive …. Mutants stir similar anxieties .. . If mutants were to acquire social and political privilege, then humans in the films (antagonists who are all white men with economic and social power) could lose their place in the social hierarchy” (200). By showing how some of the most influential components of social hierarchies are performed, mutants present audiences with recurring opportunities to reflect on the fragility of questions of societal inclusion and exclusion of minoritized groups, regardless of whether they are identified by race, sexual orientation, or gender performance.

On an interpersonal level, passing affects mutant relationships, especially when there is a discrepancy in the ability to pass between the parties. Mystique references this discrepancy when she notes to her adoptive brother that “pretty mutations, or invisible ones like yours [are worthy of pride]… But if you’re a freak, you better hide” (X5, Scene 5). Raven, whose preferred name is later Mystique, is frequently annoyed with her brother Charles Xavier (Professor X), who does not seem to appreciate or acknowledge the level of harassment and prejudice she faces as a preternaturally blue, scaly figure. She is “a freak” in a way that he is not. After Beast tells her “You have no idea what I’d give to feel normal,” she replies, “Normal. Charles has never understood. He’s different, but he’s never had to hide” (X5, Scene 18). Mystique and Beast have felt the constant pressure to hide, which negatively affected their mental health. While both characters have non-normative appearances in the comics as well, they are particularly striking on screen, surrounded by regular humans that viewers can easily relate to.

After being shot in Paris, Mystique goes to the hospital for medical treatment in a normative human form. The nurse sees Mystique on the TV from earlier in the day and remarks (translated from French):

Nurse: Terrible thing… being born like that…. Can you imagine looking in the mirror and seeing that staring back at you?

(X7, Scene 24)

The nurse does not know she is describing the patient right beside her, hiding in plain sight. Mystique could be feeling many different emotions in that moment, from isolation to shame to anger, or maybe just a dulled pain from years of disparaging comments. These types of feelings can be found across all the movies in different characters as they live their lives as mutants. As noted in the quantitative results section, the most frequent mutant emotions across all thirteen films are anger, anxiety, despair, grief, pain, and fear. While everyone experiences the whole range of emotion in their lifetime, oppression increases stress which has negative health outcomes, and poor health outcomes can be further compounded by intersections of oppression (LeBlanc et al., 2015; Brown and Homan, 2023). This problem is applicable to mutants who continue to cope with the world they live in but inevitably lead less happy lives than their non-mutant counterparts.

One generally negative emotional experience is noteworthy for how it leads to strong positive bonds in mutant community: a feeling of isolation. Perhaps the most isolating experience is to believe that no one has ever felt what you are feeling. Erik (Magneto) gives voice to the pain of isolation in X-Men: First Class (2011).

Erik: I thought I was alone.

Charles (Professor X): You’re not alone. Erik, you’re not alone.

(X5, Scene 15)

Erik, who was confined as a boy in Auschwitz during the Holocaust, never realized there were other mutants until he met Charles Xavier. This part of his identity was completely isolated. He never saw other people like him, so he believed that there were none who could relate to his experience. This feeling is comparable to queer people who grow up without access to a queer community, especially in the pre-internet age, who may have thought no one had ever felt same-gender attraction before or have felt like they were a different gender. In this scene of the movie, the relief is clear on Erik’s face. It is a powerful moment that surely resonated with audiences, either queer audience members who recognize a moment from their past or heterosexual cisgender audience members who may have never connected to this emotional experience before. Erik now knows that he is not alone, and even though he will disagree with Charles greatly in the future, in that moment he found comfort in empathy and solidarity.

Isolation is one of the many harmful internal feelings mutants have about themselves; another is self-hatred, which can arise from internalizing the hatred of society towards mutants. As previously mentioned, Raven (Mystique) and Hank (Beast) are the best examples of internalized self-hatred, though Mystique is able to form a positive self-identity more quickly than Hank in the movies. By the end of X-Men: First Class (2011), Mystique has already decided not to accept Hank’s drug that will “normalize” her appearance, but Hank is still determined to take this “cure” for his mutation.

Hank: It behooves me to tell you that even if we save the world tomorrow, and mutants are accepted into society, my feet and your natural blue form will never be deemed beautiful … . We need this cure.

(X5, Scene 56)

Hank justifies his self-hatred by being a realist. He recognizes that he and Mystique are in a different category from Charles and Erik as mutants who cannot “pass.” For Charles, acceptance in society is the ultimate goal, but Hank sees the shortcomings of that objective clearly. They may be accepted, but they “will never be deemed beautiful.” Their entire selves, in all their mutant beauty, will never be appreciated, only tolerated by wider society. Hank chooses to hide his mutation from his employer (the CIA), saying “You did not ask, so I did not tell” (X5, Scene 16). The line is a clear reference to the now defunct but long-standing “do not ask, do not tell” policy in the United States military that said LGBTQ military members should keep their queerness a secret, at least from the institution.

Mystique has the best mutant skillset to allow her to pass but to her, societal acceptance ultimately does not matter.

Mystique: You’re beautiful, Hank. Everything you are, you are perfect… We are different. But we should not be trying to fit into society. Society should aspire to be more like us. Mutant and proud.

(X5, Scene 56)

The phrase “mutant and proud” evokes similar rhetoric around pride parades and the Black power movement. “Gay pride,” “Black is beautiful,” “mutant and proud”—these phrases are all an expression of radical self-love in the face of a hostile society.

Another manifestation of self-acceptance in the X-Men world is the practice of self-naming. Because of the precedent of superhero codenames, mutants have the socially acceptable option of choosing a name for themselves. In *Deadpool 2*, Deadpool befriends a teen, Russell, who goes by “Firefist.” “Ooh, that’s a great name,” says Deadpool, smirking at his sexual innuendo (X11, Scene 18). A name can be a manifestation of one’s true self, as when Magneto asks an X-student, “What’s your *real* name?” and the boy replies, “Pyro” instead of his birthname, John. This parallels trans and nonbinary people who frequently choose different names from their birthnames to affirm their identity and sense of self. Another parallel can be seen to Black leaders like Malcolm X who changed their names to register their political convictions. But codenames are not always affirmational; many characters express an indifference to their alternative names, like when Wolverine/Logan and Rogue/Marie first meet.

Wolverine: So what kind of a name is Rogue?

Rogue: I do not know. What kind of a name is Wolverine?

Wolverine: My name’s Logan.

Rogue: Marie.

(X1, Scene 11)

Both characters are sheepish about their names, as if they are not certain why they have a second name. Part of that reluctance and confusion may stem from their origins in comic books, where almost all superhero characters have a birth name and a code name. In a scene shortly after, Wolverine makes fun of the names of several of the X-Men when he first meets them. However, his bemusement at their names quickly falls by the wayside as Wolverine learns about the school that houses and educates young mutants. Professor X began the school with an inclusive vision in mind.

Professor X: I have plans for this place. I mean to turn it into a real campus. A university. Not just for mutants either, for humans too. Living and working, growing together.

(X9, Scene 29)

Professor X’s ideal vision of the world is one of mutant-human intermingling, “living and working, growing together.” He participates in respectability politics as he aims to present mutants as upstanding citizens who can contribute to society. Professor X does not think mutants’ genetic difference defines them in relation to non-mutants; he believes that that difference can be bridged. His sister, Mystique (Raven) is much more hesitant towards Charles’ human-mutant utopia.

Mystique: Charles, I used to think it was going to be you and me against the world. But no matter how bad the world gets, you do not want to be against it, do you? You want to be a part of it.

(X5, Scene 60)

Mystique may be unsure of her place in “the world,” a reference to normative human society, but she recognizes Charles’ desire to assimilate. In subsequent films, Mystique forges her own path forward that is not as ideologically focused as her brother but is nevertheless concerned with saving and protecting mutants. In X-Men Apocalypse (2016) she is disillusioned with the idea of being a “hero,” but she still works to save mutants like Kurt Wagner (Nightcrawler) from a forced mutant fight ring by paying for a passport and extraction from Soviet East Germany. Kurt’s extraction from Soviet Germany and Laura’s journey to Canada in Logan (2017) parallel the underground railroad that enslaved people traveled to escape bondage in the American south. The underground railroad is just one allegory for these stories, however; another could be Jews escaping Europe during the rise of the Third Reich. Once again, the mutant metaphor proves highly versatile.

Mystique is primarily interested in direct action to promote mutant wellbeing, but her frequent partner in crime, Magneto, is in many ways more similar to her brother, Professor X, since they are both interested in “the world” of non-mutants. Professor X and Magneto care about society, but the former wants to be a part of it while the latter wants a mutant revolution.

Magneto: This society will not accept us. We form our own.

(X5, Scene 64)

Magneto is a mutant separatist, in contrast to Professor X’s assimilationist tendencies. Both of their reactions are in response to a hostile world that fears mutants’ genetic differences, and both of their plans require a high degree of effort on the part of their fellow mutants. To join the X-Men is to present the most respectable mutant face possible while going on difficult missions and doing promotional public acts like saving astronauts and meeting the president (*X-Men: Dark Phoenix*). To join Magneto’s Brotherhood of Mutants is to fight, plan terrorist attacks, and face constant pushback. Both paths are daunting and require ideological buy-in. It would be difficult for a mutant to join the X-Men if they did not have hopes for a mutant-human society, and it would be equally difficult to separate from non-mutant relationships without buying into a vision for mutant greatness.

Mutants are tied together by their shared genetic identity, but like any group of people they vary in their presentation and views. As we have seen, some mutants are able to “pass,” which is deeply worrying for their human antagonists who feel their position in the social hierarchy threatened, like white supremacists, antisemites, and racists. Passing mutants engage with society in part because they are able to, unlike mutants such as Mystique and Beast, who know that acceptance and assimilation into the hierarchy is not a possibility for them. But mutants vary again in their response to not passing. Beast goes to extremes to try to pass despite his appearance, while Mystique possesses a surprising self-confidence. Unfortunately, Mystique’s defiantly positive self-expression is the exception not the rule for mutants, as many struggle with isolation, self-hatred, and uncertainty about their place in society. Thus, Professor X and Magneto offer opposing visions of society, with Professor X looking to eliminate discrimination and prejudice by changing hearts and minds so mutants may safely live among non-mutants, whereas Magneto wants to form a separate mutant society. The internal group politics of mutants are readily transposable to real-world minoritized groups, such as a Black Lives Matter activist circle, a queer co-op, or an ethnic minority family, where disagreements are always plentiful because of individual variations in opinion, approach, and world outlook. But what makes X-Men films unusual for summer blockbusters is their exploration of these themes through the symbol of genetics. On a movie screen, viewers from diverse othered populations first see an assortment of mutants with visually dazzling powers, and then ultimately, perhaps unexpectedly, find themselves reflected on screen.

## Conclusion

The X-Men films demonstrate the power of blockbuster cinema to translate genetic privacy concerns and intersecting social issues of prejudice and bias to the big screen. While the movies promote diversity to appeal to the global movie marketplace, their conception of diversity can be thin and U.S. centric. Mutants in the X-Men properties always represent the Other. Their otherness stems from their mutated “X-gene” which can separate them from their “human” social group and/or create a sense of mutant solidarity. The non-mutant majority in X-Men movies frequently retaliates against mutants out of fear and hatred through discriminatory legislation, military and police violence, imprisonment, and unethical experimentation. In the face of such prejudice, mutants suffer the emotional consequences of being othered, while still finding ways to create joy and hope.

Genetic difference is the core of mutant identity, but what makes the X-Men films so compelling is their weaving of genetics in with many tropes of oppression. The mutant metaphor is extremely versatile, and any group that has faced oppression will find something relatable in the mutant experience. The two primary reference groups in the films are African Americans (racially coded visuals, excessive force used against mutants, abuse by medical institutions, allusions to the Black Power movement, a mutant underground railroad), and LGBTQ+ people (“passing”, coming out, genetic determinism, chosen family, allusion to Gay Pride parades). While we identified these categories separately, the films make no such distinctions, rather blending together different metaphors and blurring the specificity of minoritized subjects under the category of a genetic other.^5^

## Data Availability

The original contributions presented in the study are included in the article/Supplementary Material, further inquiries can be directed to the corresponding author.

## Footnotes

1
*X-Men*, 2000.

2 Representative Webster Barnaby, Florida State House, 10 April 2023.

3 Social critique may be unusual in summer blockbusters, but it is by no means unique, as has been demonstrated by the astounding success of *Barbie* (2023) in promulgating a feminist message.

4 In the comics, Storm is from Kenya. The ease with which *X-Men: Apocalypse* relocates her childhood to Egypt is further testament to the movies’ sense of the fungibility of difference.

5 The authors would like to thank their colleagues in Vanderbilt's Center for Genetic Privacy and Identity in Community Settings, especially Ellen Wright Clayton, Claire Sisco King, and Morgan Johnson, for their comments on a draft of this article.
