# Supplementary material for: Genetics in the X-Men film franchise: mutants as allegories of difference
Source: Front Genet. 2024 Jan 3;14:1331905. doi: 10.3389/fgene.2023.1331905 (PMC10882630; doi:10.3389/fgene.2023.1331905)
Supplement: Supplementary file 1 [file DataSheet1.PDF]

Supplementary Material

1 Supplementary Data

1.1 Complete Codebook

Code	*Definition*	*N*
**Character Demographics**		
Magneto’s brotherhood (only)	Scenes that exclusively feature mutants who are part of the Brotherhood of Mutants that is led by Erik Lehnsherr (Magneto).	8
Mutants (only)	Scenes that exclusively feature people with the (fictional) “X gene,” which grants them a variety of special powers.	160
Non-mutants (only)	Scenes exclusively featuring humans with no “X-gene” or mutation.	22
X-Men (only)	Scenes that exclusively feature students or teachers from Xavier's School for Gifted Youngsters, in either their capacity at school or out in the field as “X-Men”.	72
**Genetic Themes**	Touches upon genetics in some way.	13
Bio-banks	The depiction of genetic data repositories, whether held by medical institutions, corporations, or government.	4
Biological Warfare/Bioterrorism	The use of genetics for warfare or terrorism.	14
Cloning	The use of genetic information to make identical copies of an organism.	3
Deep time	The depiction of vast eons during which evolutionary change occurs; often contrasted with the more comprehensible span of historical time or individual lives.	0
Eugenics	Depictions of efforts to control a human population by encouraging or discouraging desirable or undesirable heritable traits.	4
Evolution	A focus on evolutionary changes, whether as a result of natural selection or via genetic interventions.	19
Punctuated equilibrium	An evolutionary theory that there are long periods of time with minimal/no change followed by short periods of significant change leading to speciation; e.g., mutants did not exist before the 20th century.	7
Experimentation	Experimental procedures on human subjects or mutants, whether performed in a scientific institution or by rogue scientists. This code encompasses technological enhancements used by humans.	64
Extinction	References to a species--usually, humanity--going out of existence.	25
Forensics	Instances of genetics used for gathering evidence or to diagnose a condition after the death of the organism.	1

Genealogy	Use of genetics to trace ancestry or parentage.	6
Genetic condition	Any trait, illness, with a genetic component, e.g. Alsheimer's, ALS (5-10% genetic).	9
Genetic engineering	The use of technology to modify one's genetic makeup for therapeutic purposes or to enhance an organism's characteristics.	7
Genetic screening	The depiction of programs that screen for genetic conditions. Instances of characters using genetic screening to eliminate or select for the X-gene.	2
Longevity	Scenes that refer to genetic causes of extended life spans or efforts to extend life via genetic engineering.	30
Mutation	Instances of random or induced genetic mutation.	55
Nature vs. nurture	Scenes where the contributions of nature vs. nurture to a character's identity are discussed.	3
Posthumanism	The idea that genetic changes in humans have resulted in the creation of a superior new species.	16
Radiation	Scenes that suggest that radiation causes or increases mutation.	9
Science talk	Bogus dialogue that sounds scientific enough to lend credibility to a new technology, experimental procedure, or discovery.	33
Speciation	The evolution of a species into a different species.	16
X-gene	Reference to the (fictional) gene that supposedly codes for mutations; often an allegory for Dean Hamer's so-called "gay gene".	8
**LGBTQ+**	Scenes that suggest an analogy between discrimination against mutants and queer discrimination.	10
Closet	Mutants who conceal their identity; a suggested analogy with the "closet" queer people hide in, pretending to be straight or cisgender.	32
Cure	Search or desire for a cure for mutant difference; an attempt to eliminate or silence the X-gene. Allegory for trying to "cure" people of the "disease" of homosexuality.	34
Difference	Scenes that emphasize mutants' difference from normative identities.	22
Discrimination against (LGBTQ+)	Scenes in which mutants suffer from discrimination, including individual, institutional, or governmental forms.	35
Homophobia	Fear of or aversion to homosexuality, gay people, or the larger LGBTQ+ community.	5
Isolation	Belief that you are the only one who is different, that you are alone in your experience.	34
Name	A name change, or objection to a name. Allegory for self-naming, choosing name/identity to present to the world.	30
Outing	Where someone is exposed as a mutant. Similar to exposing someone's queerness without their consent.	29

Passing	Mutants that have no visible markings of mutation, particularly in scenes where this dimension is foregrounded. A mutant who is able to "pass" as a non-mutant.	52
Queer relationships	Characters who are in queer relationships.	13
**Mutant Attitudes and Affects**	The attitudes, opinions, affects, and perspectives that mutants have about themselves and mutant-kind.	0
Anger	Resentment at unfair treatment or general lot in life as a mutant; includes moments when mutants lose control of their emotions, causing their powers to become destructive.	121
Assimilationist	Belief that the best course of action is to assimilate mutants into normal society.	30
Emotional stress	Living as a mutant causes anxiety, despair, grief, suffering, pain, or other forms of emotional strain.	118
Fear (mutants')	Fear of being persecuted, hunted. Fear of one's powers, often generalized to fear of one's own identity.	120
Hope & faith	Expressions of hope in the future and faith in the good of mutants and humans.	43
Insecurity	Doubts about one's self-worth; self-loathing; failure to accept one's unique self as valuable.	38
Mutant and proud	A strong self-belief; characters who embrace their identity and take pride in being a mutant.	42
Neanderthals	The denigration of homo sapiens as a lower species, whether or not they use specific words like homo sapiens, neanderthals, or cavemen.	24
Privacy	The presence, absence, or desire for privacy.	7
Privacy (desired)	Scenes where mutants or humans long for privacy or value their privacy, including when mutants hide from persecution.	17
Privacy (violated)	Scenes where privacy is violated, including by reading others' minds without their permission.	89
Self-control	Moments when mutants are at peace with their identities as different; moments of pleasure in their talents, serenity.	21
Us (mutants) against Them	Belief that mutants can only rely on one another; defiance of the world's prejudice. The tendency to divide the world into those who are like us and those who are Others and therefore against us; the belief that it is kill or be killed.	58
**Race**	Scenes that suggest an analogy between discrimination against mutants and racial discrimination.	16
Discrimination against (race)	Scenes in which mutants suffer from discrimination, especially when emphasizing visual signs of difference, including skin color.	41
Excessive force	Scenes where police, military, or others respond to the threat of a mutant with overwhelming force. May include "shoot first" reactions.	29

Harassment	Verbal abuse like slurs or other forms of harassment directed at mutants.	8
Revolution	Belief in the necessity of revolutionary action to throw off the effects of oppression.	15
Separatism	Belief that mutants are better off with their own kind; suggests parallels with Black Power, black nationalism, and other movements advocating the creation of a separatist society.	12
Slavery	Enslaving mutants, references to mutants as property, less than human.	8
Underground railroad	When mutants attempt to escape from a mutant-hostile state to a mutant-friendly one.	13
White supremacy	Scenes that emphasize "normal" humans as superior to mutants.	2
**Social Factors**		
Adolescence	Focus on the effects of genetic mutation or medical interventions on the young after puberty; scenes featuring adolescent characters.	80
Apocalypse	References to a real or potential apocalypse, an end of the world; apocalyptic imagery.	21
Autonomy	Focus on respecting on or infringing upon the decision-making capacities of individuals. May refer to the loss of personal sovereignty.	79
Childhood	Focus on the effects of genetic mutation or medical interventions on pre-adolescent young people; scenes featuring child characters.	51
Disability/ableism	A focus on bodily ability or disability as it relates to genetics but not in regard to the special abilities of mutants, e.g. Professor Xavier.	23
Educational institutions	Scenes that refer to schools; identify Xavier's School for Gifted Youngsters as either indulging in liberal brainwashing, or on the positive side, as supportive and nurturing.	64
Family	A general reference to family, not identified as specifically biological or chosen.	20
Family (biological)	Depictions of traditional/biological families.	121
Family (chosen)	Finding belonging in a group someone chooses to join outside of their biological family.	110
Gender	Scenes where questions of gender are highlighted, including scenes of gender bias.	58
Holocaust	Scenes that are reminiscent of the holocaust or allude to a new holocaust, a genocide.	34
Interdependence	Affirmation of the bond that unites us all, mutant and humans. The need for the support of a community. The understanding that we are all interrelated and dependent on one another for survival.	57

Nazis	Explicit or implicit references to Nazis and their experimentation on human subjects and eugenic measures; includes other references to German or Polish motifs or background.	12
Religion	Depiction of religion or spirituality.	17
Religion (+)	Positive depictions of religious practice or identity, or of religion's effect on society.	14
Religion (-)	Negative depictions of religions or religious attitudes, or of religion's effect on society.	15
Remembrance	Acknowledgment of people who have passed away, whether mutant or not. Can be allegorically connected to Holocaust remembrance, lives lost in the fight for civil rights, or the aids epidemic.	53
Sacrifice	An individual or a group must give something up for the greater good. Possibly as a consequence of the inappropriate use of genetics or for the benefit of positive scientific efforts.	26
Science	Depiction of science.	4
Science (+)	Science or scientists are depicted in a positive way.	16
Science (-)	Science or scientists are depicted in a negative way.	32
Sexuality	Expressions of sexuality.	93
State institutions	State run institutions, including any government, military, officials.	54
Legislation	Bills/laws being discussed, passed or rejected by elected officials, e.g., U.S. congress.	10
Military	Any depiction of military action, such as soldiers, generals, captains, the president acting as commander in chief.	78
**Society's View/Treatment of Mutants**	How society at large (mainly non-mutants) view and act towards mutants.	0
Admiration	Admiration, reverence, a sense of amazement or awe from non-mutants towards mutants.	17
Allies	People who are mutant-friendly; fellow travelers; supporters of the minoritized mutant community.	31
Conspiracy	Beliefs (true of not) that mutants are plotting against humanity.	10
Dangerous	Mutants are seen as posing a threat to themselves or others.	50
Fear (non-mutants')	Fear or panic related to the mutants and their capacities whether known or unknown.	92
Hostility	A generalized attitude of hatred, resentment, disgust, uneasiness, or other negative affects toward mutants.	74
Imprisonment	Jailing or imprisonment of mutants.	85
Mind control	Belief (true or not) that mutants can control the thoughts of others. Parallels the notion that LGBTQIA populations possess an outsized influence on popular culture and the young and impressionable (grooming).	4

Oppression	Laws, regulations, or other actions by the government, corporations, or civic groups that oppress selected groups, such as mutant registration.	27
Secrecy	Belief that mutants are hiding their mutation for nefarious reasons.	3
Surveillance	Spying on, recording, or watching mutants.	28
Us (humans) against Them	The tendency to divide the world into those who are like us and those who are Others and therefore against us; the belief that it is kill or be killed. Mutants are the Others.	14
Violence	Overt violence toward mutants.	60
Warfare	Action scenes or discussion of a war between humans and mutants.	44
